# Comments on ‘*In vitro* culture of cynomolgus monkey embryos beyond early gastrulation’

**DOI:** 10.1093/jmcb/mjz108

**Published:** 2019-12-21

**Authors:** Huaixiao Ma, Hongmei Wang, Ping Zheng, Lei Li

**Affiliations:** 1 State Key Laboratory of Genetic Resources and Evolution, Kunming Institute of Zoology, Chinese Academy of Sciences, Kunming 650223, China; 2 State Key Laboratory of Stem Cell and Reproductive Biology, Institute of Zoology, Chinese Academy of Sciences, Beijing 100101, China; 3 Institute for Stem Cell and Regeneration, Chinese Academy of Sciences, Beijing 100101, China; 4 Institute of Zoology, University of Chinese Academy of Sciences, Chinese Academy of Sciences, Beijing 100049, China; 5 Primate Research Center, Yunnan Key Laboratory of Animal Reproduction, Kunming Institute of Zoology, Kunming 650223, China; 6 Center for Excellence in Animal Evolution and Genetics, Chinese Academy of Sciences, Kunming 650223, China

The fertilized egg undergoes several cleavage divisions to form the blastocyst in the oviduct. The blastocyst then is transferred to the uterus to initiate implantation, and trophectoderm-derived cells interact with the maternal uterus. The dialogue between mother and embryo is prerequisite for the following embryonic development because a disturbance of this process mainly account for 60% pregnancy loss in human early post-implantation (Emiliani et al., 2005). After implantation, the embryo initiates the milestone event—gastrulation—during which multipotent epiblast cells (EPI) undergo an orchestrated process of morphogenetic cellular movement and differentiation. Through gastrulation, three germ layers (ectoderm, mesoderm, and endoderm) are specified to establish the body plan. Because the embryo is engulfed by maternal tissues and inaccessible, the direct observations and manipulations are obstructed. Currently, the knowledge on human early post-implantation development remains largely unknown.

Mouse early post-implantation development has been extensively investigated to understand the events preceding and following gastrulation in mammals (Arnold and Robertson, 2009). However, the timing of these events and embryonic morphology are obviously distinct between the rodents and the primates. For example, unlike the cup-shaped egg cylinder of mouse embryos, human or monkey embryos form a bilaminar disc structure after implantation. In 2016, two groups cultured human embryos *in vitro* for 12–13 days post-fertilization (d.p.f.) and highlighted the self-organizing properties of human embryos without the support of maternal tissues in the very beginning of early post-implantation development (Deglincerti et al., 2016; Shahbazi et al., 2016). Due to ethical constraints, the human embryo culture system may be inappropriate to address many critical questions of human early post-implantation development, such as gastrulation that occurs around 14 days (E14). Monkeys have been considered as a reliable model to study human early development. In the recent publication entitled ‘In vitro culture of cynomolgus monkey embryos beyond early gastrulation’, we established a system for the culture of non-human primate embryos *in vitro* up to 20 d.p.f. (Ma et al., 2019).

Due to the scarcity of monkey pre-implantation embryos, we firstly optimized an *in vitro* culture (IVC) system for mouse blastocysts to develop to early gastrulation based on previous methods (Hsu, 1972; Bedzhov et al., 2014). The morphology and expression patterns of the lineage markers in the IVC embryos were in good concordance with the *in vivo* mouse embryos. Comparing with the previous protocols, the optimized IVC system supported blastocysts into the formation of egg cylinder at a higher efficiency (36.4% ± 1.9%). Then, we further optimized the IVC system for monkey embryo culture and experienced that rat serum was beneficial to the succedent growth of monkey embryos. Our IVC system allowed cynomolgus monkey embryos to grow *in vitro* for up to d.p.f. 20. Strikingly, we observed a disc-like structure in the monkey IVC embryos under the optical microscope at d.p.f. 13–14. The appearance of the disc-like structure allowed us to calculate the successful rate of the IVC system (~27.7% ± 3.2%). Notably, the size of IVC embryo dramatically increased (~ 3 times) from d.p.f. 15–16 to d.p.f. 19–20, mimicking the growing of *in vivo* monkey embryos at the similar stages. Our results showed that many critical processes of monkey *in vivo* early post-implantation development occurred in the IVC embryos, such as specification of epiblast and hypoblast, formation of amnion cavity and yolk sac cavity, and differentiation of early primordial germ cells (PGCs).

**Figure 1:**
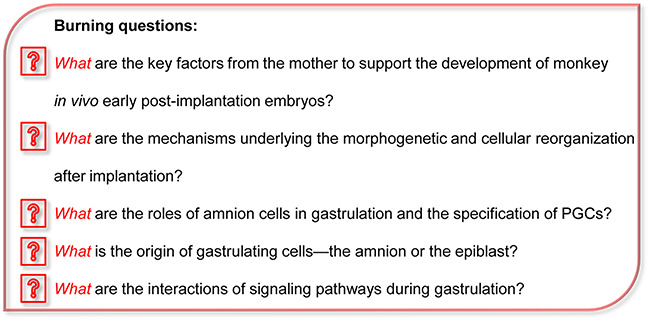
Burning questions for the future prospects.

Next, we employed this system to investigate the gastrulation, a mysterious event in primates. The d.p.f 13–14 IVC embryos exhibited key characteristics of gastrulation: appearance of potential gastrulating cells, formation of primitive streak, and establishment of anterior–posterior (AP) axis. The gastrulating cells were confirmed by the staining with the specific antibodies for OCT4 and T (Nakamura et al., 2016). The expression of T^+^/OCT^+^ was firstly observed in the amnion cells of IVC embryos at d.p.f. 11–12. Afterwards, the T^+^/OCT^+^ cell clusters seemed to emerge simultaneously at two populations: the majority located at the dorsal amnion and the minority located between EPI and VE. At d.p.f. 15–16, the T^+^/OCT4^+^ cells were predominantly present between the EPI and VE, whereas few T^+^/OCT4^+^ cells were observed at the amnion. These results suggest that gastrulating cells might have migrated down from the amnion or originate from the epiblast, which is distinct from the previous reports in mouse (Arnold and Robertson, 2009). The gastrulating cells also expressed VIMENTIN, a marker for epithelial–mesenchymal transition (EMT). The establishment of the AP axis was further validated by the gradient expression pattern of OTX2 among the cells of VE in the d.p.f. 15–16 IVC embryos. At genome-wide transcriptome of single-cell level, the IVC embryos had the similar gene expression profiles and cell types as the *in vivo* counterparts. For example, the IVC embryos included several gastrulation-related cell types, such as early gastrulating cells and late gastrulating cells 1 or 2 (E-Gast and L-Gast1 or L-Gast2) and expressed gastrulation-related genes T, OTX2, and EOMES. It is noteworthy that neural crest-like, forebrain-like, and neural groove-like structures were also observed in the monkey IVC embryos at d.p.f. 19, similar to human embryos at E17–19 (Carnegie stage 8) (O'Rahilly and Muller, 1981). Altogether, we provide strong evidence to suggest that the monkey IVC embryos self-organize beyond gastrulation without the support from the mother.

Our study also provides important information to understand the molecular characteristics and key regulators of early post-implantation embryogenesis in primates. We were the first to identify the molecular characteristics of amnion cells with expression of HOXD3, WNT6A, SPNS2, and PDZRN4. Furthermore, we revealed a close relationship between amnion cells and PGCs/gastrulating cells, suggesting that amnion cells might play crucial roles in the specification of PGCs and gastrulating cells, probably reflecting the difference between the primates and the rodents (Arnold and Robertson, 2009; Saitou and Yamaji, 2012). In addition, we observed that the activity of Wnt/β-catenin signal pathway was heterogeneous in EPI, VE, and gastrulating cells in post-implantation monkey embryos, suggesting that this pathway plays important roles in gastrulation in the primates. Combined with spatiotemporal transcriptomic analysis (Peng et al., 2016), it would be intriguing to explore the localization of specific cell types in the primate early embryos. More studies will aim to investigate the roles of these key regulators and pathways, and the origins of the specific cell types during primate early embryonic development.

Interestingly, another group reported that monkey embryos could be cultured up to d.p.f 20 *in vitro* by a method modified from human embryo culture system (Niu et al., 2019). Many key findings in our study were reproducibly observed in Niu et al. (2019). Although these studies open new avenues and provide the enriched resource to explore the dynamics and key regulators during early post-implantation development in the primates, further optimization of these monkey IVC systems is needed, e.g. through a 3D culture system closely mimicking the maternal environment of embryos (Tam, 2019). Combined with CRISPR/Cas9 gene editing and long-term living image tracing, the system of IVC monkey embryos would be helpful in scrutinizing vital cellular mechanisms and signaling interactions during early post-implantation development, probably most related with human early embryogenesis (Figure 1).

*[The work was supported by the National Key R&D Program of China (2018YFC1004500 and 2017YFC1001401), the Strategic Priority Research Program of the Chinese Academy of Sciences (XDA16020700), and `Light of West China' Program of Chinese Academy of Sciences.]*
